# Structural analysis of socioeconomic factors and school jet lag in traumatic dental injury among children

**DOI:** 10.1590/1807-3107bor-2025.vol39.005

**Published:** 2025-01-13

**Authors:** Veruska Medeiros Martins BERNARDINO, Larissa Chaves Morais de LIMA, Érick Tássio Barbosa NEVES, Matheus de França PERAZZO, Saul Martins de PAIVA, Ana Flávia GRANVILLE-GARCIA

**Affiliations:** (a) Universidade Estadual da Paraíba – UEPB, School of Dentistry, Department of Dentistry, Campina Grande, PB, Brazil.; (b) Universidade Federal de Goiás – UFG, School of Dentistry, Department of Dentistry, Goiânia, GO, Brazil.; (c) Universidade Federal de Minas Gerais – UFMG, School of Dentistry, Department of Oral Health for Children and Adolescents, Belo Horizonte, MG, Brazil.

**Keywords:** Sleep, Chronobiology Disorders, Latent Class Analysis, Child, Socioeconomic Factors

## Abstract

The objective of this study was to analyze the directions by which school jet lag is associated with traumatic dental injury in children, evaluating direct and indirect effects of socioeconomic factors and sleep. A representative, population-based, cross-sectional study was conducted with 739 schoolchildren eight to ten years of age. Parents/guardians answered a sociodemographic questionnaire, the Sleep Disturbance Scale for Children and the Circadian Energy Scale. Four examiners underwent training and calibration exercises for the diagnosis of traumatic dental injury (K > 0.80) using the criteria proposed by Andreasen (2007). Descriptive analysis was followed by structural equation modeling to determine direct and indirect associations between the variables incorporated into the theoretical model. School jet lag [standardized coefficient (SC): -0.238, 95%CI: -0.390–0.087], income (SC: -0.151, 95%CI: 0.0010–0.292), and number of residents in the home (SC: -0.109, 95%CI: -0.212–0.007) were directly associated with traumatic dental injury, whereas sleep disturbances and schooling of the parents/guardians exerted an indirect effect. Sociodemographic factors and school jet lag were associated with traumatic dental injury in children eight to ten years of age.

## Introduction

Traumatic dental injury (TDI) is a public health problem that is highly prevalent among children eight to ten years of age (14 to 22%).^1,2^ TDI affects teeth, supporting structures, and oral mucosa, exerting a negative impact on the esthetics, function, and socialization of children and, consequently, on oral health-related quality of life.^3^

Excessive daytime sleepiness is one of the factors that may be associated with TDI^2^. Sleep is part of the circadian cycle of all human beings, affecting physiological and behavioral features. The sleep-awake cycle in a 24-hour period is regulated by the suprachiasmatic nucleus located in the hypothalamus of the central nervous system. These circadian rhythms are also called the “biological clock”.^4^ The influence of the circadian cycle results in a morning, intermediate, or evening chronotype. Any difference between the chronotype of an individual and his or her work or study schedule resulting in a deficit in sleep hours during the week is called “jet lag”.^5^

The timing of of the star of school and irregular sleep hours can cause a circadian misalignment in children, resulting in “school jet lag” (SJL).^6^ Irregular sleep on school days that is compensated by children on weekends demonstrates that SJL has negative effects on physical and mental development, as it is a predictor of chronic sleep deficit.^7^ This sleep deficit is associated with a greater number of falls and injuries in children^8^ and may exert an influence on the occurrence of TDI. However, the pathway by which SJL is associated with TDI has not yet been established. Therefore, the conceptual hypothesis of the present study is that SJL exerts a direct effect on TDI and can mediate indirect associations with sleep disorders.

Sleep disorders may be associated with injuries in children,^8^ as sleep deficit alters motor abilities and cognition, which can influence the occurrence of TDI. This association may be indirect and mediated by SJL and needs to be investigated so that TDI prevention and intervention measures can be taken in oral health services.

Sociodemographic factors have been the targets of investigation in numerous studies, but the pathways by which these factors are related to TDI remain unclear.^9-11^ Income is a socioeconomic indictor of oral health outcomes,^10,11^ but the pathways that influence the association between income and TDI have not yet been established.^12^ Moreover, the schooling of parents/guardians is also a sociodemographic indicator of oral health status in children.^13^ The number of residents in the home is another sociodemographic aspect that has been little^12^explored and the pathways by which the number of residents in the home is associated with TDI have not yet been defined.

Therefore, the aim of the present study was to analyze the pathways by which SJL is associated with traumatic dental injury in children, evaluating the direct and indirect effects of socioeconomic factors and sleep.

## Methods

### Ethical considerations

This study received approval from the Human Research Ethics Committee of *Universidade Estadual da Paraíba* (certificate number: 10514619.2.0000.5187). A cross-sectional study was conducted with 739 pairs of parents/guardians and schoolchildren eight to ten years of age at public and private schools in the city of Campina Grande, Brazil, which has a Human Development Index of 0.72 and Gini Index of 0.5859.^14^

### Sample calculation

The city of Campina Grande has 73 public schools and 58 private schools with a total of 23,592 students, corresponding to 30% of the population of the city.^15^ Nine public schools and 14 private schools were randomly selected for the present study. Next, a simple sampling procedure was used at each of the selected schools considering the proportion of students in each administrative district of the city. The sample size was calculated for analytical comparison between two independent proportions using the G* Power software program, version 3.1 (Franz Faul, Universitat Kiel, Germany), considering a 95% significance level and 5% acceptable rate of error. Estimates for the calculation were based on the pilot study, in which the prevalence of TDI in children with and without sleep disorders was respectively 20% and 10%. These data determined a sample of 398 children. A design effect of 1.6 was applied to increase the variation in the sample and 20% was added to compensate for possible dropouts, resulting in a desired sample of 769 children eight to ten years of age.

### Eligibility criteria

Children with no mental, physical, sensorial, or behavioral problems and no need for special education or attention reported by the teachers were included in the study. Children who wore orthodontic appliances, those with special needs and those who parents did not live in the same house with them for at least six months and were therefore not able to provide information on the child’s sleep habits or level of disposition were excluded.

### Pilot study

A pilot study was conducted with 30 children (15 from public schools and 15 from private schools) to test the methods. The results demonstrated that the methods were adequate. The participants in this phase were not included in the main study.

### Collection of non-clinical data

The parents/guardians signed a statement of informed consent and the children signed a term of assent prior to the clinical examinations and completion of the sociodemographic questionnaire, Sleep Disturbance Scale for Children (SDSC), and Circadian Energy Scale (CIRENS) for the analysis of the child’s chronotype. The sociodemographic questionnaire addressed the child’s sex, classroom hours (morning or afternoon classes), monthly family income on Brazilian currency (R$), parent’s/guardian’s schooling and number of residents in the home. The SDSC presented a Cronbach’s alpha of 0.93, demonstrating satisfactory internal consistency. This scale evaluates sleep disorders and behaviors in the previous six months through 26 items, each with five response options. The sum of the scores attributed to each domain determines the absence/presence of sleep disorders. The cutoff point for the scale (39 points) was determined in the validation study conducted in Brazil. Scores ≥ 39 points indicate the presence of sleep disorders and scores < 39 indicate the absence of sleep disorders.^16^

The child’s chronotype was determined using a validated scale: CIRENS. This scale is used to evaluate energy level in different periods of the day (morning, afternoon and evening) and classifies the individual as the morning, intermediate or evening type. The difference between the morning and evening scores determines a single value between -4 and +4 corresponding to the chronotype.^17^ Children with the evening chronotype who studied in the morning were considered to have SJL. Children with the evening chronotype go to sleep late and have difficulty waking up in the morning. This results in an imbalance in the circadian cycle caused by the difference between the functioning of the organism (hours of sleep) and school hours, which characterizes the presence of jetlag. Children with the morning or afternoon chronotypes were not considered to have SJL regardless of the time of day in which they studied (morning or afternoon).^18,19^

### Collection of clinical data

Prior to the examinations, four examiners underwent training and calibration exercises for the diagnosis of TDI. Cohen’s Kappa coefficients for intra-examiner agreement (Kappa: 0.89–0.90) and inter-examiner agreement (Kappa: 0.81–0.88) revealed substantial reliability (K > 0.80). The examinations were performed in a reserved room at the school during classroom hours with the child in the sitting position. The examiners used personal protective equipment and an head LED lamp (Petzl Zoom head lamp, Petzl America, Clearfield, USA). The intraoral examinations were performed with the aid of sterile mouth mirrors (PRISMA, São Paulo, Brazil), sterile WHO probes (OMS-621-Trinity, Campo Mourão, Brazil) and gauze to dry the teeth. For the classification of TDI, the following criteria were used: absence of trauma, enamel fracture, enamel + dentin fracture, complicated crown fracture, extrusive luxation, lateral luxation, intrusive luxation and avulsion.^20^ Discoloration, combined traumas and restoration due to trauma were also investigated. Only the maxillary and mandibular incisors and canines were inspected for TDI.

Anthropometric data were collected. Weight was measured on a Tanita scale (Model UM080W). The child stood barefoot on the scale and had no objects in his hands, pockets or on his head. After the first reading, the child was instructed to step off the scale and step on again two more times. The mean of the three readings was used for analysis. Height was measured using a stadiometer positioned on a firm, flat surface with no rug or carpet. The child stood with the back to the support, barefoot, feet together, arms alongside the body and gazing forward. The movable arm was slid along the support until touching the top of the child’s head. After the first reading, the child was asked to leave the original position and return to it two more times for further readings. The mean of the three readings was used for analysis. Body mass index was calculated as weight (kg) divided by height (m) squared (kg/m^2^).

### Statistical analysis

Descriptive statistics were performed for the characterization of the sample with the aid of SPSS for Windows (version 25.0, IBM Inc, Amonk, USA) (absolute and relative frequencies for categorical variables; mean and standard deviation [SD] for continuous quantitative variables). Structural equation modeling was then performed using the Mplus software program, version 8.8, to evaluate direct and indirect associations between the latent variables in the theoretical model of the study, adopting a 95% confidence interval (CI).

The conceptual model was based on the results of previous studies^1,2,7,8^ or a plausible association when not found in previous studies. A summary model was initially planned for evaluating associations between TDI and SJL. The following cutoff points were used to determine the goodness of fit of the model: Comparative Fit Index (CFI > 0.90), standardized root mean square error of approximation (RMSEA < 0.06) and standardized root mean square residual (SRMR < 0.08 for acceptable fit).^21^

## Results

A total of 739 pairs of parents/guardians and children eight to ten years of age participated in the study, corresponding to a response rate of 96%. Losses occurred due to absences on the days scheduled for the examinations three consecutive times and refusals to participate. Table 1 shows the prevalence of TDI (16.2%). Table 2 shows the structural equation model and goodness-of-fit criteria. The model demonstrated a good fit: CFI = 0.955, RMSEA = 0.026; SRMR = 0.042.

**Figure 1:**
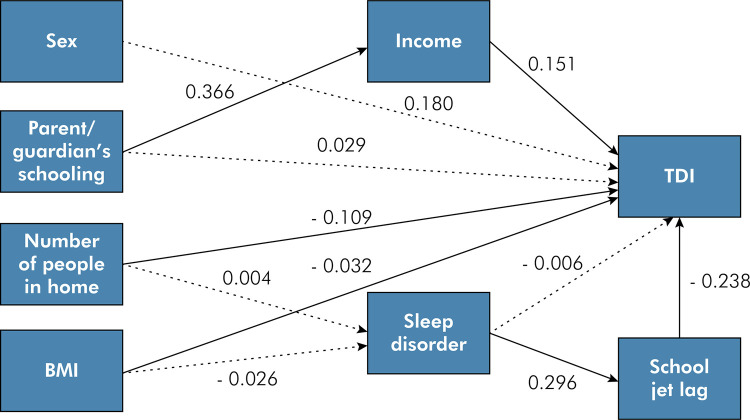
Theoretical model of the study with standardized coefficients.

**Table 1:** Description of variables used in the structural model.

Variables	Mean (SD)	Frequency	
Continuous variables			
Income (R$)	1742.68 (2304.52)	n	%
Number of residents in the home	4 (1.29)		
Sleep disorder	43.47 (12.05)		
BMI	18.2 (3.65)		
Height	137.69 (9.00)		
Weight	35.0 (10.18)		
Categorical variables			
TDI			
Yes		120	16.2
No		619	83.8
Sex			
Female		369	49.9
Male		370	50.1
Parent’s/guardian’s schooling			
> 8 years of study		419	56.9
≤ 8 years of study		318	43.1
Skin color			
White		255	34.6
Non-White		483	65.4
Child’s position in family			
Youngest		340	46.6
Oldest		275	37.7
Middle		115	15.8
School jet lag			
Yes		133	18.1
No		601	81.9

**Table 2:** Standardized estimated effects of the structural equation model.

Variables	Standardized coefficient (95%CI)	Standard error	p-value
TDI on			
Sex	0.018 (-0.092 – 0.128)	0.056	0.74
Income	0.151 (0.010 – 0.292)	0.072	0.03
Parent’s/guardian’s schooling	0.029 (-0.091 – 0.149)	0.061	0.63
Number of residents in home	-0.109 (-0.212 – 0.007)	0.052	0.03
School jet lag	-0.238 (-0.390 – 0.087)	0.077	0.002
Sleep disorders	-0.006 (-0.113 – 0.101)	0.055	0.91
BMI	-0.032 (-0.151 – 0.088)	0.061	0.60
Sleep disorders			
BMI	-0.026 (-0.103 – 0.052)	0.040	0.51
Number of residents in home	0.004 (-0.750 – 0.083)	0.040	0.91
Income			
Parent’s/guardian’s schooling	0.366 (0.285 – 0.446)	0.041	< 0.01
School jet lag			
Sleep disorders	0.296 (0.207 – 0.385)	0.045	< 0.01
Overall fit indices	95%CI	Index	
CFI	-	0.955	
RMSEA	0.000-0.051	0.026	
SRMR	-	0.042	

TDI: traumatic dental injury; CFI: Comparative Fit Index; RMSEA: root mean square error of approximation; SRMR: standardized root mean square residual.

Families with a higher monthly income (standardized coefficient = 0.151), a greater number of residents in the home (standardized coefficient = -0.109) and the presence of school jet lag (standardized coefficient = -0.238) had a greater risk of occurrence of TDI. Parent’s/guardian’s schooling exerted an indirect effect on the occurrence of TDI mediated by income. Sleep disorders exerted an indirect effect on the occurrence of TDI mediated by school jet lag.

## Discussion

The present study evaluated direct and indirect factors associated with TDI in children eight to ten years of age. The conceptual hypothesis was confirmed – SJL was directly associated with TDI and mediated the indirect association between sleep disorders and TDI. These findings are important and can assist parents/guardians in encouraging healthy living habits in children, such as maintaining adequate sleep hygiene^8^. This study also highlights the importance of the early identification of children’s chronotype so that they attend school in the period when they have more disposition, consequently reducing sleep deficit and related problems, such as accidental injuries, including TDIs.

The prevalence of TDI in the present study was high and similar to data described in previous Brazilian studies involving the same age range.^1^ Despite its prevalence, TDI is a neglected condition that often leads to functional and esthetic problems that can exert a considerable negative impact on oral health-related quality of life, especially in individuals in the growing phase.^22,23^

The prevalence of TDI was higher in children from families with a higher monthly income. In this study, income directly influenced the occurrence of TDI and mediated the indirect influence of parent’s/guardian’s schooling on TDI. Divergent results are found in the literature regarding the association between income and TDI.^9,24^ Children with a higher income have access to more goods and recreational services.^9^ Even under adult supervision, if they do not use protection equipment, such as a mouthguard, they can suffer injuries, including TDIs. In agreement with these results, a previous study found that children with a low socioeconomic status – even those who reported high athletic activities and participation in organized sports – participated less in physical activities compared to children with a medium socioeconomic level.^25^

The use of protection equipment should be encouraged during the practice of sports and recreational activities and such activities should be performed in safe environments. It is necessary to create programs that encourage the practices of sports with the creation of safe public recreational environments near homes as well as the prevention of accidents during these activities for children in all social groups. The schooling of parents/guardians was directly associated with family income and indirectly associated with TDI. This direct relationship is explained by the fact that income is the result of one’s professional occupation.^12^ Therefore, this finding shows the influence of socioeconomic status on the occurrence of TDI in the present investigation.

Another socioeconomic aspect explored in the present study was the number of residents in the home, which exerted a direct influence on TDI. This association may be explained by the fact that smaller families are able to dedicate more attention and supervision to children during their sports and recreational activities. Few studies have investigated this association^12,13^. This result is important, as it points out the vulnerability of larger families and the greater difficulty such families have in preventing TDIs. This aspect merits the attention of the health field and prevention policies.

School “jet lag” (SJL) was directly associated with TDI and mediated the indirect association between sleep disorders and TDI. SJL is the consequence of poor sleep quality and insufficient sleep quantity.^5^ The pathway by which SJL affects TDI is related to the weekly difference in hours of sleep and its consequences for children’s general health. Previous studies found that sleep deprivation was associated with bicycle accidents and falls at home and school in children and adolescents.^26,27^

In the present investigation, sleep disorders were indirectly associated with TDI and this influence was mediated by SJL. A previous study showed that sleep deprivation was not associated with TDI in adolescents, as this condition needs to be chronic in order to exert an effect on tooth injuries.^27^ Individuals with chronic sleep deprivation have other systemic conditions, such as psychological disorders, which can increase their susceptibility to accidents and falls, making them more vulnerable to TDIs.^27^ Furthermore, individuals with short or irregular sleep in early childhood are at greater risk of injuries when reaching school age,^8^ which supports the present findings that chronic sleep deficit and consequently SJL have the greatest impact on TDI.

One of the limitations of the present investigation is the possible recall bias on the answers to the sleep questionnaire (SDSC) and CIRENS. However, internal validity was ensured by the use of validated instruments, diagnostic criteria established in the literature, the calibration of the examiners and the creation of a theoretical model based on the literature. One of the strength of this study was the external validity through the careful selection of the study sample representative of the population, which facilitates the generalization of the results to similar circumstances and populations. Another strength was the statistical analysis using structural equation modeling, which enabled the understanding of the factors that exert an influence on TDI from a standpoint that has not been investigated in previous studies.

The present investigation offers unprecedented results that are important for the planning of public policies directed at children eight to ten years of age, such as educational actions to prevent traumatic dental injuries with an emphasis on adequate sleep habits and class hours, as sleep disorders are a common risk factor for various diseases. The findings can also contribute to health education and promotion actions at schools for parents/guardians directed at psychosocial aspects.

## Conclusion

Family income, number of residents in the home, and school jet lag exerted a direct effect on the prevalence of TDI in children eight to ten years of age. Moreover, school jet lag mediated the indirect association between sleep disorders and TDI and family income mediated the indirect association between parent’s/guardian’s schooling and TDI.
